# The genome sequence of the butterfly blenny, *Blennius ocellaris *Linnaeus, 1758

**DOI:** 10.12688/wellcomeopenres.23748.1

**Published:** 2025-02-19

**Authors:** Patrick Adkins, Rachel Brittain, Vengamanaidu Modepalli, Marine Biological Association Genome Acquisition Lab, Darwin Tree of Life Barcoding collective, Wellcome Sanger Institute Tree of Life Management, Samples and Laboratory team, Wellcome Sanger Institute Scientific Operations: Sequencing Operations, Wellcome Sanger Institute Tree of Life Core Informatics team, Tree of Life Core Informatics collective, Darwin Tree of Life Consortium

**Affiliations:** 1 The Marine Biological Association, Plymouth, England, UK

**Keywords:** Blennius ocellaris, butterfly blenny, genome sequence, chromosomal, Blenniiformes

## Abstract

We present a genome assembly from a specimen of *Blennius ocellaris* (the butterfly blenny; Chordata; Actinopteri; Blenniiformes; Blenniidae). The genome sequence spans 728.70 megabases. Most of the assembly is scaffolded into 24 chromosomal pseudomolecules. The mitochondrial genome has also been assembled and is 16.5 kilobases in length.

## Species taxonomy

Eukaryota; Opisthokonta; Metazoa; Eumetazoa; Bilateria; Deuterostomia; Chordata; Craniata; Vertebrata; Gnathostomata; Teleostomi; Euteleostomi; Actinopterygii; Actinopteri; Neopterygii; Teleostei; Osteoglossocephalai; Clupeocephala; Euteleosteomorpha; Neoteleostei; Eurypterygia; Ctenosquamata; Acanthomorphata; Euacanthomorphacea; Percomorphaceae; Ovalentaria; Blenniimorphae; Blenniiformes; Blennioidei; Blenniidae; Blenniinae; *Blennius*; *Blennius ocellaris* Linnaeus, 1758 (NCBI:txid195075).

## Background

**Figure 1.**
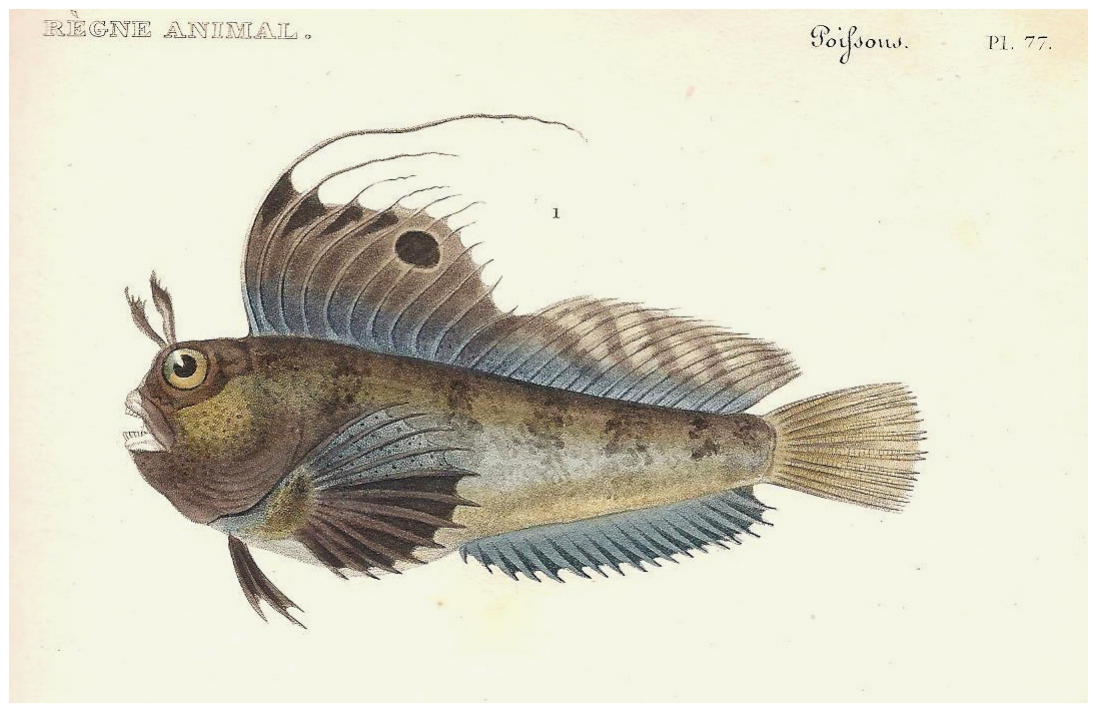
*Blennius ocellaris* illustration from Le règne animal by Georges Cuvier (1828), plate 77 (top image). Public domain image via Wikimedia Commons.

The butterfly blenny ( *Blennius ocellaris*) ( Figure 1) ( Linné & Salvius, 1758) is a small marine fish that belongs to the Blenniidae family, which is the largest family of blennies with 57 genera and 387 species ( Hastings & Springer, 2009). Blenniidae is classified under Blenniiformes, which comprises five other closely related families, including Tripterygiidae (triplefin blennies) and Blenniidae (combtooth blennies), temperate Clinidae (kelp blennies), and three largely Neotropical families (Labrisomidae, Chaenopsidae, and Dactyloscopidae). Phylogenetically, the Tripterygiidae is the sister group of all other blenniiforms, and the Blenniidae is the sister group of all remaining blennies ( Lin & Hastings, 2013).

*Blennius ocellaris* is found through northern and western Europe. It ranges from the English Channel and Irish Sea in the north, including the Isle of Man. This species can also be found in the Mediterranean Sea, parts of the Red Sea, and as far south as Morocco. It typically inhabits the subtidal zone, at depths ranging from 10 m down to over 400 m ( Whitehead, 1984). *B. ocellaris* can reach up to 20 cm in length, with one long, continuous and relatively tall dorsal fin ( Karadurmuş *et al.*, 2022). Depending on the water temperature, spawning takes place between April (Marseille) and July (England) ( Watson, 2009). Demersal and adhesive eggs are laid under mussel shells or stones and guarded by males. Larvae are planktonic and often found in shallow coastal waters ( Watson, 2009).

The genome of *Blennius ocellaris* was sequenced as part of the Darwin Tree of Life Project, a collaborative effort to sequence all named eukaryotic species in the Atlantic Archipelago of Britain and Ireland. Here we present a chromosomally complete genome sequence for *Blennius ocellaris*, based on a specimen collected from Bigbury Bay, Devon, England, UK.

## Genome sequence report

The genome of an adult specimen of *Blennius ocellaris* was sequenced using Pacific Biosciences single-molecule HiFi long reads, generating a total of 19.86 Gb (gigabases) from 1.93 million reads, providing approximately 30-fold coverage. Primary assembly contigs were scaffolded with chromosome conformation Hi-C data, which produced 90.22 Gb from 597.46 million reads. Specimen and sequencing details are given in Table 1.

**Table 1.** Specimen and sequencing data for *Blennius ocellaris*.

Project information			
**Study title**	Blennius ocellaris (butterfly blenny)		
**Umbrella BioProject**	PRJEB65187		
**Species**	*Blennius ocellaris*		
**BioSample**	SAMEA112788961		
**NCBI taxonomy ID**	195075		
Specimen information			
**Technology**	**ToLID**	**BioSample ** **accession**	**Organism part**
**PacBio long read sequencing**	fBleOce1	SAMEA112789014	gill
**Hi-C sequencing**	fBleOce1	SAMEA112789012	muscle
**RNA sequencing**	fBleOce1	SAMEA112789017	fin
Sequencing information			
**Platform**	**Run accession**	**Read count**	**Base count (Gb)**
**Hi-C Illumina NovaSeq 6000**	ERR11872533	5.97e+08	90.22
**PacBio Sequel IIe**	ERR11867193	1.93e+06	19.86
**RNA Illumina NovaSeq 6000**	ERR12035203	4.96e+07	7.48

**Figure 2.**
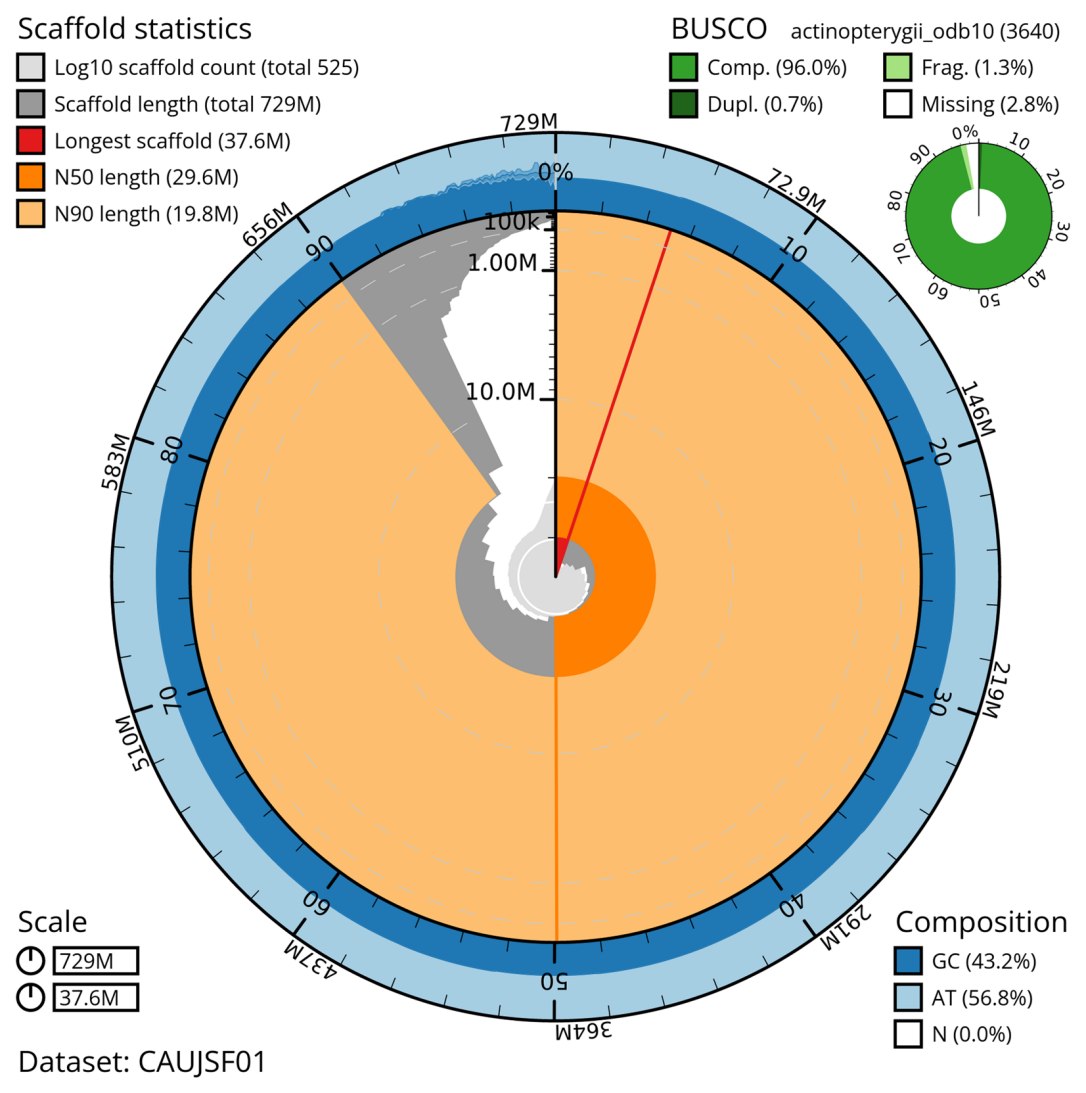
Genome assembly of *Blennius ocellaris*, fBleOce1.1: metrics. The BlobToolKit snail plot shows N50 metrics and BUSCO gene completeness. The main plot is divided into 1,000 size-ordered bins around the circumference with each bin representing 0.1% of the 728,745,255 bp assembly. The distribution of scaffold lengths is shown in dark grey with the plot radius scaled to the longest scaffold present in the assembly (37,598,765 bp, shown in red). Orange and pale-orange arcs show the N50 and N90 scaffold lengths (29,635,927 and 19,804,534 bp), respectively. The pale grey spiral shows the cumulative scaffold count on a log scale with white scale lines showing successive orders of magnitude. The blue and pale-blue area around the outside of the plot shows the distribution of GC, AT and N percentages in the same bins as the inner plot. A summary of complete, fragmented, duplicated and missing BUSCO genes in the actinopterygii_odb10 set is shown in the top right. An interactive version of this figure is available at https://blobtoolkit.genomehubs.org/view/CAUJSF01/dataset/CAUJSF01/snail.

**Figure 3.**
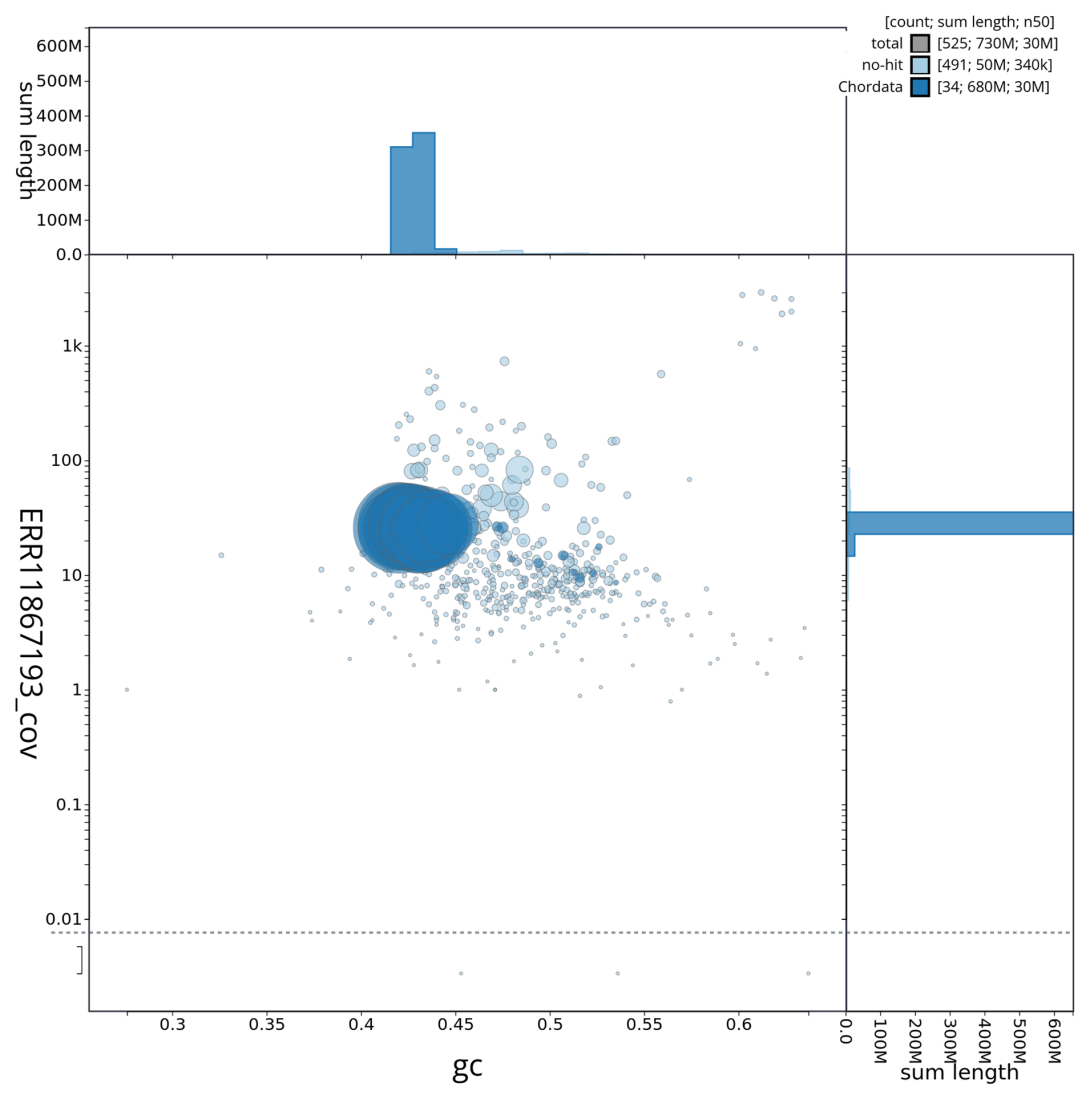
Genome assembly of *Blennius ocellaris*, fBleOce1.1: BlobToolKit GC-coverage plot. Sequences are coloured by phylum. Circles are sized in proportion to sequence length. Histograms show the distribution of sequence length sum along each axis. An interactive version of this figure is available at https://blobtoolkit.genomehubs.org/view/CAUJSF01/dataset/CAUJSF01/blob.

**Figure 4.**
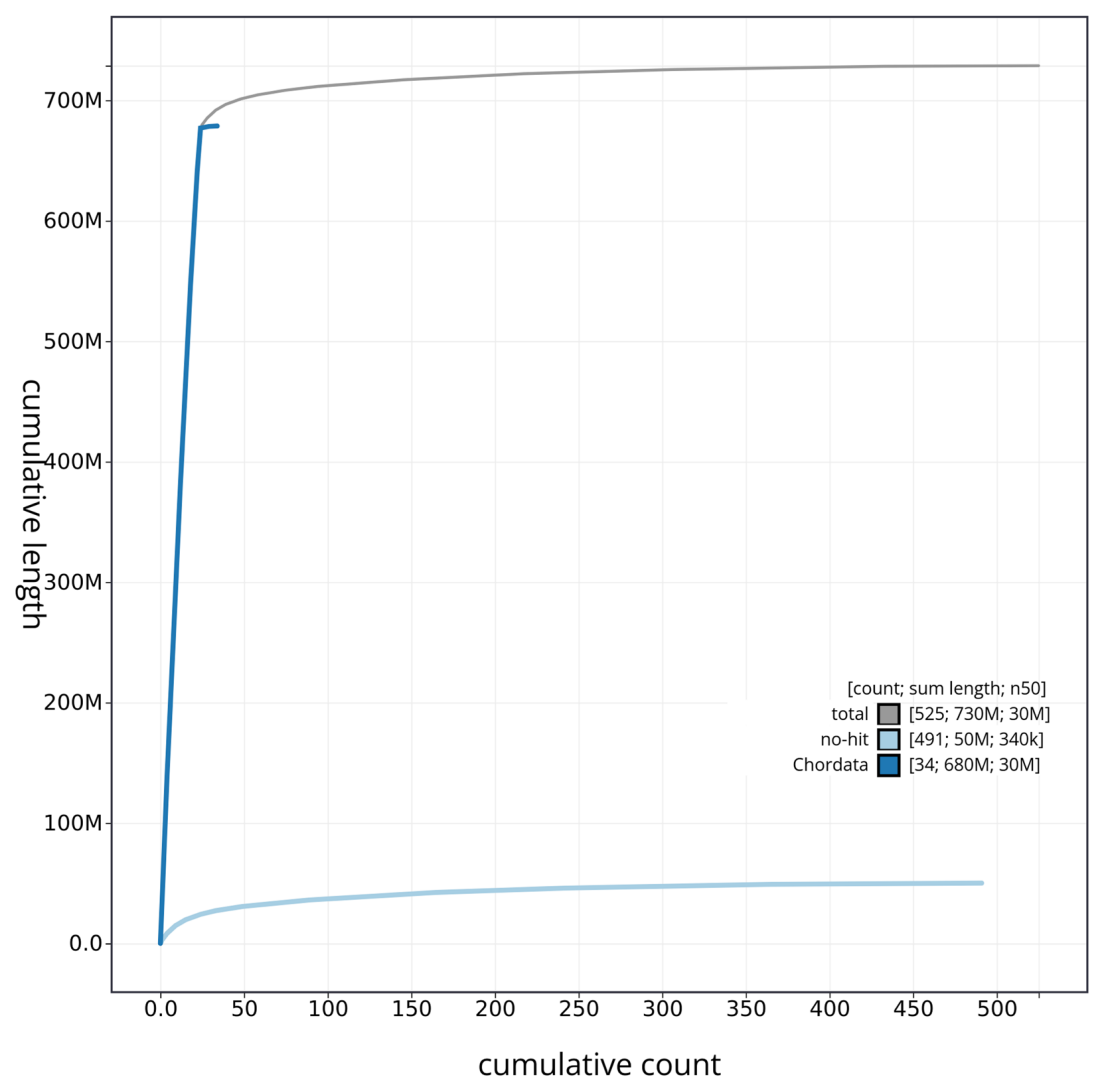
Genome assembly of *Blennius ocellaris* fBleOce1.1: BlobToolKit cumulative sequence plot. The grey line shows cumulative length for all sequences. Coloured lines show cumulative lengths of sequences assigned to each phylum using the buscogenes taxrule. An interactive version of this figure is available at https://blobtoolkit.genomehubs.org/view/CAUJSF01/dataset/CAUJSF01/cumulative.

**Figure 5.**
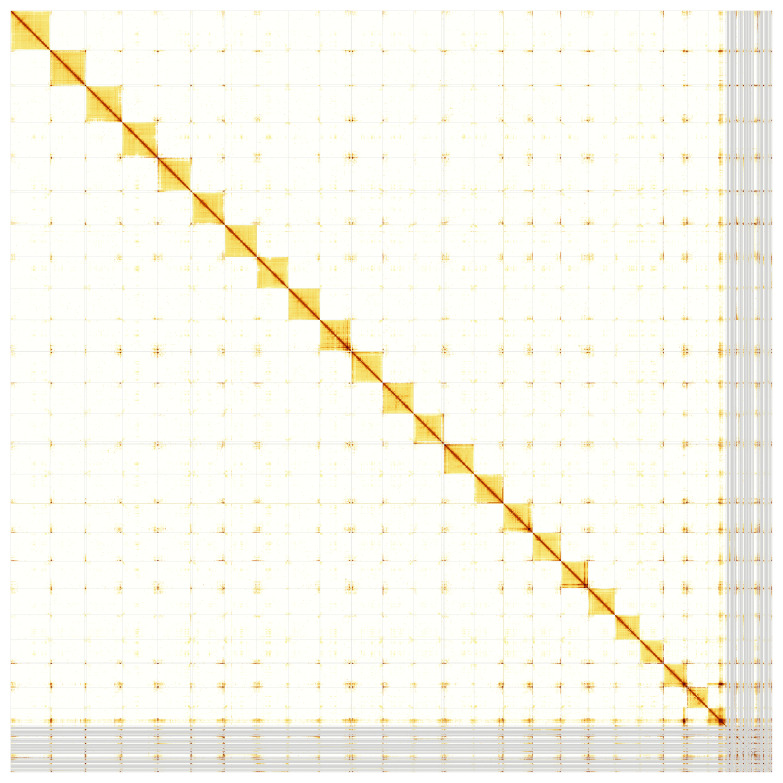
Genome assembly of *Blennius ocellaris*: Hi-C contact map of the fBleOce1.1 assembly, produced from the PretextView map. Chromosomes are shown in order of size from left to right and top to bottom.

Manual assembly curation corrected 53 missing joins or mis-joins, reducing the scaffold number by 1.87%, The final assembly has a total length of 728.70 Mb in 524 sequence scaffolds with a scaffold N50 of 29.6 Mb ( Table 2). The total count of gaps in the scaffolds is 1,625. The snail plot in Figure 2 provides a summary of the assembly statistics, while the distribution of assembly scaffolds on GC proportion and coverage is shown in Figure 3. The cumulative assembly plot in Figure 4 shows curves for subsets of scaffolds assigned to different phyla. Most (94.08%) of the assembly sequence was assigned to 24 chromosomal-level scaffolds. Chromosome-scale scaffolds confirmed by the Hi-C data are named in order of size ( Figure 5; Table 3). While not fully phased, the assembly deposited is of one haplotype. Contigs corresponding to an alternate haplotype have also been deposited. The mitochondrial genome was also assembled and can be found as a contig within the multifasta file of the genome submission.

**Table 2.** Genome assembly data for *Blennius ocellaris*, fBleOce1.1.

Genome assembly		
Assembly name	fBleOce1.1	
Assembly accession	GCA_963422515.1	
*Accession of alternate haplotype*	*GCA_963422475.1*	
Span (Mb)	728.70	
Number of contigs	2,150	
Number of scaffolds	524	
Longest scaffold (Mb)	37.6	
Assembly metrics *		*Benchmark*
Contig N50 length (Mb)	1.1	*≥ 1 Mb*
Scaffold N50 length (Mb)	29.6	*= chromosome N50*
Consensus quality (QV)	51.8	*≥ 40*
*k*-mer completeness	primary: 90.16%; alternate: 86.69%; combined: 98.56%	*≥ 95%*
BUSCO **	C:96.0%[S:95.2%,D:0.7%], F:1.3%,M:2.8%,n:3,640	*S > 90%*, *D < 5%*
Percentage of assembly mapped to chromosomes	94.08%	*≥ 90%*
Sex chromosomes	Not identified	*localised homologous pairs*
Organelles	Mitochondrial genome: 16.5 kb	*complete single alleles*

* Assembly metric benchmarks are adapted from Rhie *et al.* (2021) and the Earth BioGenome Project Report on Assembly Standards September 2024. ** BUSCO scores based on the actinopterygii_odb10 BUSCO set using version 5.3.2. C = complete [S = single copy, D = duplicated], F = fragmented, M = missing, n = number of orthologues in comparison. A full set of BUSCO scores is available at https://blobtoolkit.genomehubs.org/view/CAUJSF01/dataset/CAUJSF01/busco.

**Table 3.** Chromosomal pseudomolecules in the genome assembly of *Blennius ocellaris*, fBleOce1.

INSDC accession	Name	Length (Mb)	GC%
OY730201.1	1	37.6	42.0
OY730202.1	2	33.29	42.0
OY730203.1	3	34.44	43.0
OY730204.1	4	33.95	42.5
OY730205.1	5	31.33	43.0
OY730206.1	6	31.21	43.0
OY730207.1	7	30.59	42.5
OY730208.1	8	30.31	43.0
OY730209.1	9	30.11	42.5
OY730210.1	10	29.78	42.5
OY730211.1	11	29.97	42.5
OY730212.1	12	29.64	42.0
OY730213.1	13	26.57	42.5
OY730214.1	14	28.65	43.0
OY730215.1	15	28.32	42.5
OY730216.1	16	27.95	44.0
OY730217.1	17	27.09	43.0
OY730218.1	18	25.87	43.0
OY730219.1	19	25.63	43.0
OY730220.1	20	23.95	43.5
OY730221.1	21	21.94	43.5
OY730222.1	22	22.5	44.0
OY730223.1	23	19.8	43.5
OY730224.1	24	16.46	44.5
OY730225.1	MT	0.02	42.5

The estimated Quality Value (QV) of the final assembly is 51.8 with *k*-mer completeness of 98.56% for the combined assemblies (90.16% for the primary and 86.69% for the alternate haplotype). The assembly has a BUSCO v5.3.2 completeness of 96.0% (single = 95.2%, duplicated = 0.7%), using the actinopterygii_odb10 reference set ( *n* = 3,640).

## Methods

### Sample acquisition and DNA barcoding

An adult specimen of *Blennius ocellaris* (specimen ID MBA-220429-002A, ToLID fBleOce1) was collected from Bigbury Bay, Devon, England, UK (latitude 50.26, longitude –3.95) from the RV Sepia on 2022-04-29. The specimen was collected and identified by Patrick Adkins (Marine Biological Association) and identified by Rachel Brittain (Marine Biological Association) and preserved on dry ice.

The initial identification was verified by an additional DNA barcoding process according to the framework developed by Twyford *et al.* (2024). A small sample was dissected from the specimens and stored in ethanol, while the remaining parts of the specimen were shipped on dry ice to the Wellcome Sanger Institute (WSI). The tissue was lysed, the COI marker region was amplified by PCR, and amplicons were sequenced and compared to the BOLD database, confirming the species identification ( Crowley *et al.*, 2023). Following whole genome sequence generation, the relevant DNA barcode region is also used alongside the initial barcoding data for sample tracking at the WSI ( Twyford *et al.*, 2024). The standard operating procedures for Darwin Tree of Life barcoding have been deposited on protocols.io ( Beasley *et al.*, 2023).

### Nucleic acid extraction

The workflow for high molecular weight (HMW) DNA extraction at the Wellcome Sanger Institute (WSI) Tree of Life Core Laboratory includes a sequence of procedures: sample preparation; sample homogenisation, DNA extraction, fragmentation, and clean-up. In sample preparation, the fBleOce1 sample was weighed and dissected on dry ice ( Jay *et al.*, 2023). Gill tissue was homogenised using a PowerMasher II tissue disruptor ( Denton *et al.*, 2023).

HMW DNA was extracted using the Automated MagAttract v2 protocol ( Oatley *et al.*, 2023a). DNA was sheared into an average fragment size of 12–20 kb in a Megaruptor 3 system ( Bates *et al.*, 2023). Sheared DNA was purified by solid-phase reversible immobilisation ( Oatley *et al.*, 2023b), using AMPure PB beads to eliminate shorter fragments and concentrate the DNA. The concentration of the sheared and purified DNA was assessed using a Nanodrop spectrophotometer and Qubit Fluorometer using the Qubit dsDNA High Sensitivity Assay kit. Fragment size distribution was evaluated by running the sample on the FemtoPulse system.

RNA was extracted from fin tissue of fBleOce1 in the Tree of Life Laboratory at the WSI using the RNA Extraction: Automated MagMax™ *mir*Vana protocol ( do Amaral *et al.*, 2023). The RNA concentration was assessed using a Nanodrop spectrophotometer and a Qubit Fluorometer using the Qubit RNA Broad-Range Assay kit. Analysis of the integrity of the RNA was done using the Agilent RNA 6000 Pico Kit and Eukaryotic Total RNA assay.

### Hi-C sample preparation

Tissue from muscle tissue of fBleOce1 sample was processed for Hi-C sequencing at the WSI Scientific Operations core, using the Arima-HiC v2 kit. In brief, 20–50 mg of frozen tissue (stored at –80 °C) was fixed, and the DNA crosslinked using a TC buffer with 22% formaldehyde concentration. After crosslinking, the tissue was homogenised using the Diagnocine Power Masher-II and BioMasher-II tubes and pestles. Following the Arima-HiC v2 kit manufacturer's instructions, crosslinked DNA was digested using a restriction enzyme master mix. The 5’-overhangs were filled in and labelled with biotinylated nucleotides and proximally ligated. An overnight incubation was carried out for enzymes to digest remaining proteins and for crosslinks to reverse. A clean up was performed with SPRIselect beads prior to library preparation. Additionally, the biotinylation percentage was estimated using the Qubit Fluorometer v4.0 (Thermo Fisher Scientific) and Qubit HS Assay Kit and Arima-HiC v2 QC beads.

### Library preparation and sequencing

Library preparation and sequencing were performed at the WSI Scientific Operations core.

***PacBio HiFi***

At a minimum, samples were required to have an average fragment size exceeding 8 kb and a total mass over 400 ng to proceed to the low input SMRTbell Prep Kit 3.0 protocol (Pacific Biosciences, California, USA), depending on genome size and sequencing depth required. Libraries were prepared using the SMRTbell Prep Kit 3.0 (Pacific Biosciences, California, USA) as per the manufacturer's instructions. The kit includes the reagents required for end repair/A-tailing, adapter ligation, post-ligation SMRTbell bead cleanup, and nuclease treatment. Following the manufacturer’s instructions, size selection and clean up was carried out using diluted AMPure PB beads (Pacific Biosciences, California, USA). DNA concentration was quantified using the Qubit Fluorometer v4.0 (Thermo Fisher Scientific) with Qubit 1X dsDNA HS assay kit and the final library fragment size analysis was carried out using the Agilent Femto Pulse Automated Pulsed Field CE Instrument (Agilent Technologies) and gDNA 55kb BAC analysis kit.

Samples were sequenced using the Sequel IIe system (Pacific Biosciences, California, USA). The concentration of the library loaded onto the Sequel IIe was in the range 40–135 pM. The SMRT link software, a PacBio web-based end-to-end workflow manager, was used to set-up and monitor the run, as well as perform primary and secondary analysis of the data upon completion.

***Hi-C***

For Hi-C library preparation, DNA was fragmented using the Covaris E220 sonicator (Covaris) and size selected using SPRISelect beads to 400 to 600 bp. The DNA was then enriched using the Arima-HiC v2 kit Enrichment beads. Using the NEBNext Ultra II DNA Library Prep Kit (New England Biolabs) for end repair, a-tailing, and adapter ligation. This uses a custom protocol which resembles the standard NEBNext Ultra II DNA Library Prep protocol but where library preparation occurs while DNA is bound to the Enrichment beads. For library amplification, 10 to 16 PCR cycles were required, determined by the sample biotinylation percentage. The Hi-C sequencing was performed using paired-end sequencing with a read length of 150 bp on an Illumina NovaSeq 6000 instrument.

***RNA***

Poly(A) RNA-Seq libraries were constructed using the NEB Ultra II RNA Library Prep kit, following the manufacturer’s instructions. RNA sequencing was performed on the Illumina NovaSeq 6000 instrument.

### Genome assembly, curation and evaluation

***Assembly***

The HiFi reads were assembled using Hifiasm ( Cheng *et al.*, 2021) with the --primary option. Haplotypic duplications were identified and removed using purge_dups ( Guan *et al.*, 2020). The Hi-C reads were mapped to the primary contigs using bwa-mem2 ( Vasimuddin *et al.*, 2019). The contigs were further scaffolded using the provided Hi-C data ( Rao *et al.*, 2014) in YaHS ( Zhou *et al.*, 2023) using the --break option. The scaffolded assemblies were evaluated using Gfastats ( Formenti *et al.*, 2022), BUSCO ( Manni *et al.*, 2021) and MERQURY.FK ( Rhie *et al.*, 2020).

The mitochondrial genome was assembled using MitoHiFi ( Uliano-Silva *et al.*, 2023), which runs MitoFinder ( Allio *et al.*, 2020) and uses these annotations to select the final mitochondrial contig and to ensure the general quality of the sequence.

***Assembly curation***

The assembly was decontaminated using the Assembly Screen for Cobionts and Contaminants (ASCC) pipeline (article in preparation). Manual curation was primarily conducted using PretextView ( Harry, 2022), with additional insights provided by JBrowse2 ( Diesh *et al.*, 2023) and HiGlass ( Kerpedjiev *et al.*, 2018). Scaffolds were visually inspected and corrected as described by Howe *et al.* (2021). Any identified contamination, missed joins, and mis-joins were corrected, and duplicate sequences were tagged and removed. The process is documented at https://gitlab.com/wtsi-grit/rapid-curation (article in preparation).

***Assembly quality assessment***

The Merqury.FK tool ( Rhie *et al.*, 2020), run in a Singularity container ( Kurtzer *et al.*, 2017), was used to evaluate *k*-mer completeness and assembly quality for the primary and alternate haplotypes using the *k*-mer databases ( *k* = 31) that were computed prior to genome assembly. The analysis outputs included assembly QV scores and completeness statistics.

The genome was analysed within the BlobToolKit environment ( Challis *et al.*, 2020) and BUSCO scores ( Manni *et al.*, 2021) were calculated.

Table 4 contains a list of relevant software tool versions and sources.

**Table 4.** Software tools: versions and sources.

Software tool	Version	Source
BlobToolKit	4.2.1	https://github.com/blobtoolkit/ blobtoolkit
BUSCO	5.3.2	https://gitlab.com/ezlab/busco
bwa-mem2	2.2.1	https://github.com/bwa-mem2/ bwa-mem2
Gfastats	1.3.6	https://github.com/vgl-hub/ gfastats
Hifiasm	0.19.5-r587	https://github.com/chhylp123/ hifiasm
HiGlass	1.11.6	https://github.com/higlass/higlass
Merqury	MerquryFK	https://github.com/ thegenemyers/MERQURY.FK
Minimap2	2.24-r1122	https://github.com/lh3/minimap2
MitoHiFi	3	https://github.com/marcelauliano/ MitoHiFi
PretextView	0.2	https://github.com/sanger-tol/ PretextView
purge_dups	1.2.5	https://github.com/dfguan/purge_ dups
samtools	1.15.1	https://github.com/samtools/ samtools
sanger-tol/ascc	-	https://github.com/sanger-tol/ ascc
YaHS	1.2a.2	https://github.com/c-zhou/yahs

### Wellcome Sanger Institute – Legal and Governance

The materials that have contributed to this genome note have been supplied by a Darwin Tree of Life Partner. The submission of materials by a Darwin Tree of Life Partner is subject to the **‘Darwin Tree of Life Project Sampling Code of Practice’**, which can be found in full on the Darwin Tree of Life website here. By agreeing with and signing up to the Sampling Code of Practice, the Darwin Tree of Life Partner agrees they will meet the legal and ethical requirements and standards set out within this document in respect of all samples acquired for, and supplied to, the Darwin Tree of Life Project.

Further, the Wellcome Sanger Institute employs a process whereby due diligence is carried out proportionate to the nature of the materials themselves, and the circumstances under which they have been/are to be collected and provided for use. The purpose of this is to address and mitigate any potential legal and/or ethical implications of receipt and use of the materials as part of the research project, and to ensure that in doing so we align with best practice wherever possible. The overarching areas of consideration are:

•    Ethical review of provenance and sourcing of the material

•    Legality of collection, transfer and use (national and international)

Each transfer of samples is further undertaken according to a Research Collaboration Agreement or Material Transfer Agreement entered into by the Darwin Tree of Life Partner, Genome Research Limited (operating as the Wellcome Sanger Institute), and in some circumstances other Darwin Tree of Life collaborators.

## Data Availability

European Nucleotide Archive: Blennius ocellaris (butterfly blenny). Accession number PRJEB65187; https://identifiers.org/ena.embl/PRJEB65187. The genome sequence is released openly for reuse. The *Blennius ocellaris* genome sequencing initiative is part of the Darwin Tree of Life (DToL) project. All raw sequence data and the assembly have been deposited in INSDC databases. The genome will be annotated using available RNA-Seq data and presented through the Ensembl pipeline at the European Bioinformatics Institute. Raw data and assembly accession identifiers are reported in Table 1 and Table 2.

## Author information

Members of the Marine Biological Association Genome Acquisition Lab are listed here: https://doi.org/10.5281/zenodo.8382513.

Members of the Darwin Tree of Life Barcoding collective are listed here: https://doi.org/10.5281/zenodo.12158331.

Members of the Wellcome Sanger Institute Tree of Life Management, Samples and Laboratory team are listed here: https://doi.org/10.5281/zenodo.12162482.

Members of Wellcome Sanger Institute Scientific Operations: Sequencing Operations are listed here: https://doi.org/10.5281/zenodo.12165051.

Members of the Wellcome Sanger Institute Tree of Life Core Informatics team are listed here: https://doi.org/10.5281/zenodo.12160324.

Members of the Tree of Life Core Informatics collective are listed here: https://doi.org/10.5281/zenodo.12205391.

Members of the Darwin Tree of Life Consortium are listed here: https://doi.org/10.5281/zenodo.4783558.
