# Supplementary material for: The genome sequence of the butterfly blenny, Blennius ocellaris Linnaeus, 1758
Source: Wellcome Open Res. 2025 Feb 19;10:87. [Version 1] doi: 10.12688/wellcomeopenres.23748.1 (PMC11883214; doi:10.12688/wellcomeopenres.23748.1)
Supplement: Peer review history

# Reviewer report: Reviewer response for version 1

**DOI:** 10.21956/wellcomeopenres.26196.r119778

**Published:** 2025-03-24

**Authors:** Ruiqi Li

**Affiliations:** 1 University of Colorado Boulder, Boulder, Colorado, USA

Adkins *et al*. presents a high quality genome of the butterfly blenny, *Blennius ocellaris*. I only have minor comments.

 1. In the introduction, could you please write more on what biological questions this genome could help us answer? You can have a look at Li *et al*. 2024 as an example.

 2. Figure 1. Do you have a picture of the specimen you used?

Are sufficient details of methods and materials provided to allow replication by others?

Yes

Is the rationale for creating the dataset(s) clearly described?

Yes

Are the datasets clearly presented in a useable and accessible format?

Yes

Are the protocols appropriate and is the work technically sound?

Yes

Reviewer Expertise:

Genomics

I confirm that I have read this submission and believe that I have an appropriate level of expertise to confirm that it is of an acceptable scientific standard.

## References

[1] : The genome sequence of the smooth giant clam, Tridacna derasa Röding, 1798. *Wellcome Open Res* .2024;9: 10.12688/wellcomeopenres.22618.1 375 10.12688/wellcomeopenres.22618.1 39429632 PMC11490831

[2] : The genome sequence of a heart cockle, Fragum fragum (Linnaeus, 1758). *Wellcome Open Res* .2024;9: 10.12688/wellcomeopenres.21134.1 129 10.12688/wellcomeopenres.21134.1 38989474 PMC11234083

# Reviewer report: Reviewer response for version 1

**DOI:** 10.21956/wellcomeopenres.26196.r119775

**Published:** 2025-03-05

**Authors:** Mariana F. Nery

**Affiliations:** 1 State University of Campinas, São Paulo, Brazil

The manuscript presents a high-quality genome assembly for the butterfly blenny ( *Blennius ocellaris*), a marine fish species belonging to the Blenniidae family. The genome assembly is of high quality, with excellent contiguity and completeness. The method is comprehensive and well-documented, with appropriate sequencing depth (30x coverage) and validation metrics. The assembly meets or exceeds all benchmark standards for vertebrate genome assemblies. The authors provide a thorough description of the species' taxonomy, habitat, and biological characteristics.

 Minor points: the manuscript mentions RNA sequencing (RNA-Seq) data was generated, but does not clearly state how this data was used in the assembly process or if it will be used for future annotation work. No sex chromosomes were identified in the assembly, but there is no discussion about whether this reflects biological reality or technical limitations.

Are sufficient details of methods and materials provided to allow replication by others?

Partly

Is the rationale for creating the dataset(s) clearly described?

Yes

Are the datasets clearly presented in a useable and accessible format?

Yes

Are the protocols appropriate and is the work technically sound?

Yes

Reviewer Expertise:

Genomics

I confirm that I have read this submission and believe that I have an appropriate level of expertise to confirm that it is of an acceptable scientific standard.

# Reviewer report: Reviewer response for version 1

**DOI:** 10.21956/wellcomeopenres.26196.r119119

**Published:** 2025-02-24

**Authors:** Emily Giles

**Affiliations:** 1 Molecular Surveillance, Cawthron Institute, Nelson, New Zealand; 2 Universidad Austral de Chile, Valdivia, Los Ríos Region, Chile

Assemblies for the genome and mitogenome of the butterfly blend, *Blennius ocellarius*, are presented. The approximately 700Mb genome is assembled in 24 pseudo chromosomes. PacBio HiFi, 30X coverage, and HiC mapping were used to generate the assembly. Hifiasm was used for the primary assembly, haplotig duplications were removed with purge_dups, scaffolding with the HiC data was done using YaHS. Complete BUSCO scores are extremely high and duplicated, fragmented, and missing BUSCOs are very low. Overall, the assembly is very high quality, and a stringent decontamination protocol (ASCC) was used to remove contamination. This being said, the lack of details within this genome report of how ASCC works limits the reader's understanding of the decontamination process. The author's state that the ASCC publication is in process, though I believe this is the second or third genome report that I have reviewed that states the same. The documentation for ASCC on GitHub is quite limited, again leaving the reader to infer how contamination was actually removed. It would be nice to know why the authors did not just simply remove non-cordate contig hits recovered from blob tools.  Furthermore, the authors state that RNA was extracted and sequenced, but there is no mention of how this was used. One assumes this will be used for the annotation, but this is not stated in the manuscript. The authors could improve clarity on these two points.

Are sufficient details of methods and materials provided to allow replication by others?

Partly

Is the rationale for creating the dataset(s) clearly described?

Yes

Are the datasets clearly presented in a useable and accessible format?

Yes

Are the protocols appropriate and is the work technically sound?

Yes

Reviewer Expertise:

marine comparative genomics, marine population genomics

I confirm that I have read this submission and believe that I have an appropriate level of expertise to confirm that it is of an acceptable scientific standard.
